# Well Water as a Possible Source of *Waddlia chondrophila* Infections

**DOI:** 10.1264/jsme2.ME12048

**Published:** 2012-10-05

**Authors:** Francesc Codony, Mariana Fittipaldi, Esther López, Jordi Morató, Gemma Agustí

**Affiliations:** 1 Laboratori de Microbiologia Sanitària i Mediambiental (MSM-Lab), Universitat Politècnica de Catalunya, Edifici Gaia-Parc UPC, Rambla Sant Nebridi, 08222 Terrassa, Barcelona, Spain

**Keywords:** *Waddlia chondrophila*, waterborne transmission, quantitative PCR, environmental detection, infection source

## Abstract

*Waddlia chondrophila* is an emerging pathogen considered as a potential agent of abortion in humans and bovines, and is related with human respiratory disease. Despite these findings, the infection source and transmission pathways have not been identified. The evidence of growth into amoeba suggests water as a possible environmental source. The presence of *Waddlia chondrophila* was determined in drinking and well water samples (*n*=70) by quantitative PCR (Q-PCR). Positive results were observed in 10 (25%) of the 40 well samples analyzed; therefore, well water could be a potential reservoir and possible infection source of *Waddlia chondrophila* in animals and humans.

*Waddlia chondrophila* is an obligate intracellular bacterium belonging to the family *Waddliaceae* and order *Chlamydiales* and has been related to abortions in ruminants (5) and with respiratory disease and miscarriages in humans (1, 11). *W. chondrophila* DNA was detected in respiratory samples from patients with pneumonia and children with bronchiolitis, suggesting that this bacterium may also cause respiratory tract infections (8, 11). Baud *et al.* (1) carried out a prospective study which showed the first evidence that *W. chondrophila* could be implicated in human fetal death. These results demonstrated, for the first time, a strong association between the presence of *W. chondrophila*-IgG-specific antibodies and early fetal loss. *Waddlia* seroprevalence seems to be higher in women who had had recurrent and sporadic miscarriages than in women who had had uneventful pregnancies (1). Subsequent studies proved the human pathogenic potential of *W. chondrophila* (6, 7, 12). These authors showed the great capacity of this pathogen to enter and multiply rapidly within human macrophages, inducing the lysis of infected cells.

Despite these findings, the *W. chondrophila* infection source and its transmission pathways have not been identified yet. Moreover, little is known about its environmental distribution and prevalence. Some studies have showed an association between contact with animals and positive serologic results for *Waddlia*, raising its zoonotic potential (1, 5). In addition, ingestion of contaminated water, meal or milk, and sexual transmission have been suggested as other possible infection pathways (1), although is still uncertain whether infection with *W. chondrophila* occurs through any of these infection pathways. The evidence of *W. chondrophila* growth in free-living amoebae has been shown in different studies (5, 9, 14). Free-living amoebae are present worldwide (10, 16) and have been isolated from freshwater, seawater, soil, air, and human nasal mucosa (4, 17, 20). Furthermore, amoebae can colonize several kinds of water systems, they are widespread in drinking water systems (5, 10, 18), and they can endure the harsh physical and/chemical conditions of these systems, such as elevated temperature or biocides (19). The active role of free-living amoebae in the survival of different pathogens in drinking-water systems and consequently in their infectious disease transmission has been largely demonstrated (4). Microorganisms capable of survival and growth in amoebae include bacteria, viruses, and fungi (10). In the *Chlamydia* genus, various interactions between chlamydiae and amoebae may take place (5). In the case of *W. chondrophila*, different evidence has shown that free-living amoebae may serve as hosts of this pathogen (5, 9, 14). Evidence that part of the lifecycle of this microorganism is related with amoebae and that ingestion of contaminated water has been reported in the literature as a possible *Waddlia* infection pathway (1) suggest water as a possible environmental source of *W. chondrophila*, as it is observed in other amoeba-parasitic microorganisms (5, 10). To confirm this hypothesis, the presence of *W. chondrophila* was analyzed in different water samples (*n*=70) by Q-PCR.

During a 12-month period, 70 water samples were analyzed, 40 from wells and 30 from drinking systems. Samples came from different urban areas around Barcelona (Catalonia, Spain) in a surrounding area of 25 km^2^. All samples were collected from different areas and came from different sampling points and sources. At each point, 1.5 L water samples were collected aseptically in sterile plastic containers supplemented with 1.5 mL sodium thiosulphate (3% [w/v]). Samples were transported at a lower temperature of 8°C. They were received by the laboratory within 5 h and stored at 2–5°C until they were processed, always within 18 h after reception. With the aim of characterizing these water samples, conventional microbiological analyses and *W. chondrophyla* detection by Q-PCR were carried out.

Microbiological analyses were performed using standardized methods: Spanish official methods for coliform/*E. coli* detection, International Organization for Standardization (ISO) and Health Protection Agency (HPA). The following microbiological parameters were determined: *Escherichia coli* and total coliforms (http://www.boe.es/boe/dias/2009/03/31/pdfs/BOE-A-2009-5316.pdf), *Legionella* spp. (http://www.iso.org/iso/home/store/catalogue_tc/catalogue_detail.htm?csnumber=19653), *Clostridium perfringens* (http://www.hpa-standardmethods.org.uk/documents/food/pdf/F14.pdf), intestinal enterococci (http://www.iso.org/iso/home/store/catalogue_tc/catalogue_detail.htm?csnumber=14854), and colony counts at 22°C (http:www.iso.org/iso/home/store/catalogue_tc/catalogue_detail.htm?csnumber=28960).

Briefly, for total coliforms, *E. coli*, *C. perfringens* and intestinal enterococci determination, water samples (100 mL) were concentrated by filtration using a nitrocellulose membrane filter (0.45 μm pore; Merck Millipore, Billerica, Massachusetts, US). Microbiology assays for total coliforms and *E. coli* were performed on Chromocult agar plates (Merck, Madrid, Spain) supplemented with 0.0025 mg L^−1^ cefsulodin-vancomicin (Sigma Aldrich, Madrid, Spain) at 37°C for 24 h. *C. perfringens* were detected by plating in Tryptose Sulfite Cycloserine Agar and incubating in anaerobic jars for 24 h at 44°C. Presumptive colonies of *C. perfringens* were characterized biochemically on the basis of the fermentation of lactose, gelatine hydrolysis and nitrate reduction. For intestinal enterococci agar Slanetz-Bartley agar plates (Oxoid, Basingstoke, UK), were incubated at 36°C for 48 h, with further confirmation by esculin hydrolysis at 44°C for 3 h. Total aerobic microorganisms were determined by plating 0, 1 and 1 mL of different water samples on triptone soya agar (TSA, Oxoid, Basingstoke, UK), with subsequent sample incubation at 22°C for 72 h. For *Legionella* sp. determination, water samples (1 L) were concentrated by filtration, (0.45 μm nylon filter; Merck Millipore). Microbiology assays were performed on Legionella GVPC agar plates (Oxoid, Basingstoke, UK) for 240 h at 37°C, after pre-treating different sub-samples with heat (50°C for 30 min) or acid (pH 2.2 for 5 min). Presumptive *Legionella* sp. colonies were confirmed by plating presumptive colonies in Buffered charcoal yeast extract agar plates with and without cysteine (Oxoid, Basingstoke, UK).

No noteworthy microbiological differences were observed between the two types of water tested (Table 1). In addition, no significant differences were found between positive and negative well samples. The results showed good microbiological quality in all water samples in relation to the Spanish regulations regarding drinking water (http://www.boe.es/boe/dias/2003/02/21/pdfs/A07228-07245.pdf), which is a transposition of European Community rules.

*Waddlia* detection has focused on biological samples (sera, fetal samples) and on the use, for this detection, of serology, phenotypic analysis in culture media, and immune fluorescence (1, 5). This microorganism is difficult to isolate and cultivate from samples as it usually appears in low concentrations and needs restrictive culture media. Currently, PCR is an alternative to detect the presence of *W. chondrophila* through amplification of specific DNA sequences (7, 8). The evolution of conventional PCR to Q-PCR has further improved the rapid and accurate identification of molecular-based methods. Different molecular studies have shown that Q-PCR is an excellent method for *W. chondrophila* detection in biological samples (3, 7–9, 12). Moreover, the recent availability of the *W. chondrophila* genome has opened up new research perspectives (2).

For Q-PCR analysis, 1 L water was collected and concentrated by membrane filtration (0.45 μm nylon filter; Merck Millipore). Cells were resuspended in 10 mL PBS (1×, pH 7.4) and were detached from the membrane filter by vigorous vortexing for 60 s into a sterile tube containing glass beads (5-mm diameter) and sonication for 3 min in an ultrasound water bath (40W power, 40 kHz ultrasound frequency; JP Selecta, Barcelona, Spain). To obtain a pellet, centrifugation at 14,000×*g* for 5 min was performed. Subsequently, the pellet was resuspended in 200 μL PBS. DNA was extracted with an EZNA tissue DNA purification kit (Omega Bio-Tek, Norcross, Georgia, US) according to the manufacturer’s instructions. The PCR procedure was based on previous work by Goy *et al.* (8), and was performed on a LightCycler-1.5 PCR system (Roche Molecular Diagnostics, Pleasanton, US). Briefly, the reaction mixture was composed of 10 μL Fast Start Taqman Probe Master (Roche Molecular Diagnostic), 0.4 U Uracil-DNA-glycosylase (UDG, New England Biolabs, UK), 9 μL DNA sample, 0.2 μM *W. chondrophila*-specific primers WadF4 (5′-GGCCCTTGGGTCGTAAAGTTCT-3′), WadR4 (5′-CGGAGTTAGCCGGTGCTTCT-3′), which delimited a 101 bp DNA fragment of the 16S rRNA gene of *W. chondrophila*, and 0.1 μM probe WadS2 (5′-FAM-CATGGGAACAAGAGAAGGATG-BHQ-3′). The probe contained locked nucleic acids (underlined in sequence above). The Q-PCR conditions had been optimized previously (data not shown) and were as follows: one step of 2 min at 50°C to allow UDG to break down the possible contaminating amplicons, one step of 10 min at 95°C, and 50 cycles of 15 s at 95°C and 1 min at 60°C. A negative control (water PCR grade) and positive control (DNA from *W. chondrophila*) were included in each assay. Additionally, a standard curve was constructed and the quantification limit was calculated by making serial 10-fold dilutions of the initial bacteria DNA stock dilution using sterile TAE buffer to obtain the set of dilutions for the curve. *W. chondrophila* was identified in 10 (25%) of 40 analyzed well water samples. The range of concentrations found was between 2.2×10^1^ and 1.4×10^3^ copies L^−1^, the detection limit being 2.0×10^1^ copies L^−1^. Regarding the drinking water samples, all were negative for *W. chondrophila*.

Taking into account the microbial diversity in environmental samples, which show great genetic diversity, some doubts often rise about the specificity of primers and probes, so it is difficult to perform a complete validation of the results, even though genetic knowledge about *Chlamydia*-related bacteria has considerably improved during the last years. This drawback is usual in environmental microbiology. For this reason, to increase the specificity of *W. chondrophila* detection, DNA sequence analysis was performed. PCR products were recovered from the LightCycler capillaries and then were purified using a Cycle-Pure kit (Omega Biotek) and sequenced (SECUGEN, Madrid, Spain) using WadR4 primer. Subsequently, DNA sequences were identified against GenBank database with the BLAST family program package (http://blast.ncbi.nlm.nih.gov/Blast.cgi). DNA sequences obtained from PCR products were similar in all positive samples, differing only by 2–3 bp. These sequences were about 50 pb and belonged to the DNA fragment adjacent to the WadR4 primer. All of the PCR products from positive amplification contained sequences compatible with the target gene of *W. chondrophila* 2032/99, *W. chondrophila* WSU 86-1044 and *Waddlia* sp. G817. BLAST results indicated identity of 100%, 100%, and 95%, respectively, and expectation values of 7e–17, 7e–17 and 1e–09, respectively. The sequence obtained most from the PCR products was as follows: “TTAGCATCCTTCTCTTGTTCCCATGCGAAAGAACTTTACGACCCAAGGGCCA”.

In conclusion, the obtained results suggest that the hypothesis that *W. chondrophila* could have an environmental source is probable, and can be related to water. Regarding different water sources, microbiological analysis highlighted that samples without treatment, such as well water, could contain *W. chondrophila* at levels high enough to be detected by Q-PCR. On the other hand, drinking water, which showed good microbiological quality according to Spanish regulations, did not contain *W. chondrophila* or its concentration was below the analytical detection limit. Samples came from different urban areas around Barcelona. No differences in microbiological characteristics were observed between positive and negative well samples (Table 1). In addition, no differences were observed among these samples in terms of geographical distribution. *Waddlia* presence in some well water might have been caused by groundwater pollution due to human activity, as the presence of septic tanks or agriculture, as underground flow tends to clean water particles and microbial contaminants.

The presence of *W. chondrophila* in water sources, such as well water, suggests that this water source can have a direct role in bovine infection and an indirect role in human infection, because well water can be used for drinking water production. Well water could be the origin of *W. chondrophila* in other water environments such as swimming pools, spas or drinking water systems. In these systems this bacterium can find appropriate conditions to proliferate, as do *Legionellae* (13) and other bacteria of the *Chlamydia* genus (*Simkania negevensis*) (15).

To the best of our knowledge, our results are the first report to demonstrate that well water could be an important potential reservoir and a possible infection source of *W. chondrophila*. Moreover, this study is the first prospective survey to contribute to the understanding of *W. chondrophila* environmental distribution. To date, there are no similar studies of *W. chondrophila*. Nevertheless, further studies are needed to reinforce these results to establish the implication of waterborne transmission in the epidemiology of this bacterium. Additionally, it is necessary to clarify and detail the likelihood of infection by *W. chondrophila* through exposure with infected water. This knowledge could be an important step forward that may minimize exposure and subsequent *W. chondrophila* infection in bovines or humans to reduce respiratory diseases and miscarriages where this pathogen is implicated. In addition, considering these preliminary results, it is necessary to improve our knowledge about the distribution of *W. chondrophila* in other different environments, such as different water sources (swimming pools, spas, hot water in large buildings and outlet water from treatment plants) and soils.

Since this pathogen requires host cells, such as amoebae, to survive environmental conditions, further research regarding bacteria–amoebae interactions is necessary, as well as to determine the best treatment for water disinfection in order to eliminate this pathogen from the environment. In addition, another aspect of study could be the evaluation of *W. chondrophila* survival under conditions without host cells, in order to know the viability of naked *Waddlia* under these conditions.

## Figures and Tables

**Table 1:** Summary of microbiological analysis results of different water samples (*n*=70)^a^

Water sample source (*n*)	Microorganisms	Min	Max	Mean	*n^p^* (%)
Public drinking water (30)	Total coliforms (cfu 100 mL^−1^)	<10	3.00×10^2^	4.14×10^1^	25 (83.3)
	*Escherichia coli* (cfu 100 mL^−1^)	nd	nd	nd	0 (0)
	Total Aerobic bacteria (TAB) (cfu mL^−1^)	<10	8.48×10^2^	2.56×10^2^	24 (80)
Well (40)	Total coliforms (cfu 100 mL^−1^)	<10	2.50×10^3^	1.37×10^2^	23 (57.5)
	*Escherichia coli* (cfu 100 mL^−1^)	nd	nd	nd	0 (0)
	*Legionella spp.* (cfu L^−1^)^b^	nd<100	nd<100	nd<100	— (0)
	*Clostridium perfringens* (cfu 100 mL^−1^)	<10	<10	<10	1 (2.5)
	Intestinal enterococci (cfu 100 mL^−1^)	<10	<10	<10	2 (5)
	Total Aerobic bacteria (TAB) (cfu mL^−1^)	<10	2.20×10^2^	2.90×10^1^	32 (80)

a Abbreviations and symbols: *n*, number of analysed samples; *n^p^*, number of positive analysed samples; nd, not detected.

b Limit of *Legionella* detection is 100 cfu L^−1^.
